# Good Early Results Obtained with a Guided-Motion Implant for Total Knee Arthroplasty: A Consecutive Case Series

**DOI:** 10.2174/1874325001711010051

**Published:** 2017-02-24

**Authors:** Hagen Hommel, Kai Wilke

**Affiliations:** Krankenhaus Märkisch Oderland GmbH BT Wriezen, Klinik für Orthopädie, Sportmedizin und Rehabilitation, Wriezen, Germany.

**Keywords:** Osteoarthritis, Knee, Arthroplasty, Knee replacement, Gap-balancing, Extension-first technique, Balancer device, Outcome assessment

## Abstract

**Background:**

Previous studies have shown a high incidence of complications with a bi-cruciate stabilized (BCS) guided-motion total knee arthroplasty (TKA) design, which led to recent modifications of the design by the manufacturer.

**Objective:**

The current study was undertaken to assess whether the use of this TKA system with an extension-first surgical technique is associated with a similar rate of short-term adverse outcome as reported in literature.

**Material and Methods:**

This retrospective study enrolled 257 consecutive patients (257 knees) undergoing TKA for osteoarthritis of the knee, with the first 153 receiving cemented Journey BCS I implants and the remaining 104 receiving cemented Journey BCS II implants when these became available.

**Results:**

Mean follow-up time for the cohort was 24.5 ± 7.8 months (range, 12 - 36 months). There were no cases of stiffness. Incidence of iliotibial friction syndrome was considered low: three (2.0%) knees in the BCS I group and two (1.9%) in the BCS II group (p = 0.676). Five (2.5%) knees presented with mild instability in midflexion, three (2.0%) in the BCS I group and two (1.9%) in the BCS II group (p = 0.676). One patient with a BCS I implant required reoperation for aseptic loosening 23 months postoperatively. At one-year follow-up, there were no clinically relevant differences in any of the clinical outcomes.

**Conclusion:**

When used in combination with an extension-first surgical technique, good early functional results with an acceptable rate of complications were obtained with both the original and the updated Journey BCS knee implant.

## INTRODUCTION

Total knee arthroplasty (TKA), though primarily considered a successful procedure, has also been associated with post-surgical functional deficits in activities of daily living [1, 2]. To address the potential underlying causes of these deficits, several new knee implants were introduced in recent years that seek to obtain improved stability and higher flexion.

One such implant (Journey Bi-Cruciate Stabilized [BCS], Smith & Nephew, Memphis, TN, USA) recreates a specific kinematic model through the principle of guided motion [3]. It aims to improve knee kinematics by more closely approximating a normal knee with an asymmetric femoral component, polyethylene replicating 3° of tibial varus, and a medially concave and laterally slightly convex shape [4]. Guided motion is obtained via the asymmetric tibiofemoral surface geometry and cam-post design, the latter of which guides the femur to external rotation in flexion in relation to the tibia and in full extension to the screw-home mechanism. The function of both the anterior cruciate ligament and posterior cruciate ligament is replicated by the post-cam’s ability to engage posteriorly as well as anteriorly [5].

Recent data from a retrospective analysis indicated superior results with this device when compared with another guided-motion implant, the Scorpio Non-Restrictive Geometry posterior-stabilized knee system (Stryker Orthopedics, Mahwah, NJ) [6]. Patients undergoing TKA with the Journey BCS experienced statistically significantly better outcomes in the Knee Injury and Osteoarthritis Outcome Score (KOOS) subcategories of pain and quality of life, as well as in postoperative range of motion [6].

Other authors have reported a high rate of early complications following implantation of the Journey BCS [4, 5, 7]. Of specific concern was a study reporting an increased risk of postoperative iliotibial band (ITB) friction syndrome (7.2%) with this device after a mean follow-up time of 2.5 years (range, 1 – 5 years), which eventually led to a surgical release of the ITB in 2% of these subjects [5]. These authors also reported revision rates of 0.5% for tibial component loosening, 0.4% for patellar component loosening, and 0.1% for instability. They concluded that the asymmetric cam and post mechanism does not allow for the natural kinematic variability in the knee [5].

In 2011, a modified implant (Journey BSC II) was released with design features meant to improve upon the earlier model, including moving the post anteriorly and increasing its height to reduce the possibility of dislocation resulting from the cam ‘jumping’ over it. Additionally, the posterior slope was increased in the lateral compartment and the medial compartment, and the posterior lip was moved anteriorly in the medial compartment [8].

The current retrospective analysis was performed to assess whether the use of a guided-motion knee system in our clinic was associated with a short-term adverse outcome rate similar to that reported elsewhere in the literature. The secondary aim was to assess if there were any differences between the first and second generations of this implant.

## MATERIALS AND METHODS

Between June 2011 and December 2013, 257 eligible patients (257 knees; Table **1**) undergoing TKA for osteoarthritis of the knee at our medical center were consecutively enrolled. Patients underwent TKA if they had persistent knee pain that was unresponsive to conservative treatment. Patients under the age of 18 at the time of surgery, with rheumatoid arthritis or post-traumatic arthrosis, and/or who did not provide their consent, were excluded. Study data were prospectively collected during routine follow-up protocol and analyzed retrospectively thereafter. The Ethics Commission of the State Chamber of Medicine in Brandenburg has approved the study (Reg. no: AS 12(bB)/2015), and all patients provided their informed consent.

All patients received a cemented Journey BCS knee system (Smith & Nephew Inc., Memphis, TN, USA) with an oxidized zirconium-niobium articular surface. The first 153 patients (59.5%) received Journey BCS I implants, whereas the remaining 104 patients (41.5%) received Journey BCS II implants when these became available. From that point forward, only BCS II implants were used at the clinic.

All patients were operated upon by the first author using a medial parapatellar approach and an extension-first technique previously described in the literature [9]. Soft-tissue balancing was performed by first setting the extension gap with a balancer device [10] and then, where appropriate, gradually releasing the ligament to achieve a symmetrical extension gap [11-13]. The extension gap was then applied to the flexion gap. The balancer device was used to distract the femur from the proximal tibia. The rotation of the femur was adjusted based on the soft tissue tension, with an aim of achieving a rectangular flexion gap. The final bone cuts were then performed, followed by implantation of the prosthetic device. Patellar treatment consisted of removal of osteophytes, patellar denervation by electrocautery, without replacement of the patella. In none of the patients there was any need for a lateral release to correct patellar tracking. Full weight-bearing was allowed on the 3^rd^ postoperative day, beginning with two crutches and then reduced to one crutch according to the patient’s ability to balance. After their stay in the clinic, patients were sent to a rehabilitation center until they had obtained full flexion of the operated knee.

Clinical data were obtained preoperatively and at one-year follow-up. All patients with follow-up time of more than 12 months were invited to the clinic for an additional physical assessment. Knee Society Score [14] was documented preoperatively, at one year, and at the latest follow-up time. Stiffness was defined as flexion ability less than 90°. Patients were systematically assessed for the presence of ITB friction syndrome and instability at 0°, 60° and 90° of flexion. Positive findings were reported when symptoms of focal tenderness over the lateral femoral epicondyle and lateral knee pain between 20° and 80° of motion were noted [5]. Midflexion instability was determined in 60° of flexion [15]. The Western Ontario and McMasters Universities Arthritis Index (WOMAC) questionnaire [16] was documented at the latest postoperative visit.

Anteroposterior, lateral, and long-leg full weight-bearing radiographs were taken at the one-year follow-up.

Implant failure at any time following surgery was defined as removal of any implant component for any cause.

Categorical variables are presented as frequencies and percentages. Continuous data are presented as mean and standard deviation (SD). Univariate analysis was performed using the Chi-squared or the Fisher’s exact test for categorical variables, and the Student’s t-test for continuous variables. Treatment comparisons for the continuous longitudinal outcome variables were based on mixed linear models. The preoperative level was used as an explanatory variable. Two-sided tests were used throughout, and p<0.05 was considered statistically significant. Stata 12.1 (Stata Corp, College Station, TX, USA) was used for the analysis.

## RESULTS

Preoperative data and baseline data are presented in Table **1**. There were no patients lost to follow-up. Mean follow-up time for the entire cohort was 24.5 ± 7.8 months (range, 12 – 36), 28 months (range, 18 – 36 months) for those receiving BCS I, and 15 months (range, 12 – 18 months) for those receiving BCS II.

Favorable clinical results were obtained in both groups (Table **2**). There were no cases of stiffness (flexion < 90°). Five knees (1.9%) were observed mimicking ITB friction syndrome, three (2.0%) in the BCS I group and two (1.9%) in the BCS II group (p = 0.676). None of these knees required medication or surgical intervention, and were therefore considered to be of clinically marginal relevance. In addition to ITB, we observed five knees (1.9%) with mild instability in midflexion, of which three (2.0%) were in the BCS I group and 2 (2.9%) were in the BCS II group (p = 0.676).

At one-year follow-up, there were significant differences in range of motion, knee score, or function score (Table **2**). At the time of the latest follow up, knee score was slightly better for the BCS II group, but no significant differences were found for function and WOMAC scores.

The mean (± SD) postoperative hip-knee-ankle was 1.3º ± 1.1º varus. There were no cases of radiolucent lines observed.

Complications are summarized in Table **3**. One patient with a BCS I implant required reoperation for aseptic loosening 23 months postoperatively. One patient exhibited signs of infection. However, puncture showed no infection, and the knee is still in situ.

## DISCUSSION

The current study found that positive clinical results and a low rate of complications can be obtained with a guided-motion BCS knee implant used in combination with an extension-first surgical technique. These results are noteworthy for standing in contradiction to those published elsewhere in the literature with the same knee design.

Guided-motion designs have been reported to lead to excessive posterior translation of the lateral condyle and internal rotation of the tibia with increasing flexion, which in turn causes stretching of the ITB [5] and associated stiffness [8]. It was theorized that the underlying factor for these complications was the asymmetric cam and post’s role as a hard driver of posterior femoral translation and internal tibial rotation during flexion [5]. These forces were thought to prevent the attainment of natural kinematic adaptability in native knees, as well to initiate repeated involuntary ITB stretching that leads to painful traction syndrome in some cases [5].

The theory that excessive ITB elongation was related to this design feature was called into question by a recent cadaver study, which failed to establish a clear pathogenesis for this adverse outcome [8]. Cadaveric results from this analysis did provide supporting evidence that the BCS I design led to excessive tightening of the soft tissues adjacent to the knee through the mechanism of over-internal rotation and rollback, although the authors concluded that design adjustments introduced for the BCS II reduced the risk of this outcome [8].

In our retrospective series, we observed no subjects with postoperative stiffness, either with the original BCS I or updated BCS II designs. This in contrast to stiffness rates with the original BCS I design of 22.6% [7], 2.7% [6], and 2.2% [4] reported in the literature. It remains unclear whether the absence of stiffness in our series was supported by the kinematic rotational alignment obtained using an extension-first surgical technique. Femoral malrotation is known to result in anterior knee pain and stiffness [9], and may lead to an asymmetrical flexion gap with resulting flexion instability [17]. Victor *et al* have shown that surgical technique and soft tissue handling are determinants of kinematics for this particular implant [3]. The extension-first technique we employed may be more forgiving with guided-motion implants in comparison with the measured resection or tibia-first technique, the latter of which has been associated with midflexion instability in a recently published study in 226 consecutive knees [4]. The tibia-first technique may lead to femoral component malrotation in patients with preoperative deformities that result in ligamental instabilities [9, 18] and is furthermore associated with postoperative joint line elevation [4]. Both component malrotation and joint line elevation are risk factors for midflexion instability [15, 19].

Luyckx *et al* reported no differences in component rotation when employing an extension-first or adapted measured resection technique [20]. In the latter technique, femoral rotation was based on the posterior condylar line adapted according to the native rotational geometry of the distal femur using the pre-operative CT scan [20]. No postoperative clinical comparison between the two techniques was undertaken.

In the current study, we observed statistically significant differences for KS and FS between the two versions of the implant. Differences were marginal and well below the minimal clinically important differences (KS, 5 points and FS, 6 points [21]). This implies that excellent outcome can be obtained with both versions of the implant with the use of an extension-first technique.

Although we believe the follow-up period used in this study was adequate for assessing our chosen endpoints, it is clear that this also represents an inherent limitation. A next-generation knee system’s total impact, as measured in benefits and unforeseen deficits, may only become apparent with the availability of long-term results, particularly as they apply to the outcome of revision. The fact that there are only few published data with the BCS II implant, however, makes the relatively brief follow-up period employed here less of a concern. Such data represent an unmet need that may shed light on initial performance of a new device and thereby better inform clinical decision-making.

Furthermore, it was not possible to conduct a proper comparison between the BCS I and II in this analysis. Follow-up times, and other important variables, differed between the groups. A randomized clinical trial would be needed to identify relevant differences in outcome between them.

## CONCLUSION

Good early functional results with an acceptable rate of complications were obtained with both the original as well as the updated Journey BCS knee implant when used in combination with an extension-first surgical technique. Long-term follow-up studies are needed to confirm our findings.

## Figures and Tables

**Table 1:** Patient baseline characteristics.

	**Total (n = 257)**	**BSC I (n = 153)**	**BSC II (n = 104)**	**p-value**
Age [years]*	68.9 ± 6.2	69.2 ± 6.2	68.5 ± 6.2	0.411
Sex (female / male)^§^	150 / 107	90 / 63	60 / 44	0.857
BMI [kg/m^2^]*	29.1 ± 3.4	28.8 ± 3.4	29.5 ± 3.4	0.154
ASA (1 / 2 / 3)^§^	88 / 154 / 15	55 / 88 / 10	33 / 66 / 5	0.659
Preoperative KS*	25 ± 4	25 ± 5	26 ± 4	0.483
Preoperative FS*	21 ± 6	21 ± 6	21 ± 6	0.525

Presented as mean ± standard deviation*, number of observations^§^. Abbreviations: BMI = body mass index; ASA = American Society of Anesthesiologists Classification; KS = Knee Score; FS = Function Score.

**Table 2:** Clinical outcome data.

	**Total (n = 257)**	**BSC I (n = 153)**	**BSC II (n = 104)**	**p-value**
KS at 1-year follow-up^§^	90 (89 – 91)	90 (89 – 90)	91 (90 – 92)	0.035
KS at final follow-up^§^	91 (90 - 91)	90 (89 – 91)	91 (90 – 92)	0.003
FS at 1-year follow-up^§^	88 (87 – 99)	87 (86 – 88)	89 (88 – 91)	0.027
FS at final follow-up^§^	88 (87 – 89)	88 (86 – 89)	88 (86.5 – 88.9)	0.367
ROM at 1-year follow-up^§^	125 (123 – 126)	123 (121 – 124)	127 (125 – 129)	0.001
WOMAC at final follow-up^§^	23 (23 – 24)	23 (23 – 24)	23 (22 – 24)	0.359
HKA Angle*	1.3 ± 1.1	1.3 ± 1.2	1.2 ±1.0	0.740

Presented as mean (95% confidence interval) ^§^ or mean ± standard deviation*. Abbreviations: KS = Knee Score; FS = Function Score; ROM = range of motion; HKA = hip-knee-ankle.

**Table 3:** Postoperative complications.

**Complication**	**Total (n = 257)**	**BCS I (n = 153)**	**BCS II (n = 104)**
Hematoma	2 (0.8%)	1 (0.7%)	1 (1.0%)
Suspected infection	1 (0.4%)	1 (0.7%)	0 (0.0%)
Wound healing disturbance	3 (1.2%)	2 (1.3%)	1 (1.0%)
Aseptic loosening	1 (0.4%)	1 (0.7%)	0 (0.0%)
